# A novel model for predicting the prognosis of postoperative intrahepatic cholangiocarcinoma patients

**DOI:** 10.1038/s41598-023-45056-9

**Published:** 2023-11-06

**Authors:** Yinghao Lv, Hu Liu, Penghui He, Sinan Xie, Xiuchun Yin, Yunshi Cai, Hong Wu

**Affiliations:** 1 Liver Transplant Center, Transplant Center, State Key Laboratory of Biotherapy and Cancer Center, West China Hospital, Sichuan University and Collaborative Innovation Center of Biotherapy, Sichuan University, No. 37, Guo Xue Xiang, Chengdu, 610041 Sichuan Province China; 2 Ward of Liver Transplant Centre and Vascular Surgery, West China Hospital, Sichuan University, Chengdu, Sichuan China

**Keywords:** Biliary tract cancer, Liver cancer

## Abstract

Intrahepatic cholangiocarcinoma (ICC) accounts for 20% of liver malignancies with a 5-year survival rate of 35% at best with limited prognostic predictors. Lung Immune Prognostic Index (LIPI) is a novel prognostic factor in pulmonary cancers. In this study, we developed a modified prognostic model from LIPI called intrahepatic immune prognostic index (IIPI) for ICC. A retrospectively study was conducted at Liver Transplant Center of West China Hospital between January 2015 and January 2023. Hematological factors and clinical features of ICC patients were collected and analyzed. The area under curve (AUC) and optimal cuff-off of each single hematological factor was calculated. In this study, derived neurtrophil to lymphocyte ratio (dNLR), arbohydrate antigen199 (CA199) and carcinoembryonic antigen (CEA) have higher AUC values. LIPI was composed of dNLR and was further modified by combing CA199 and CEA, forming the IIPI. The IIPI consists of four grades which are None, Light, Moderate and Severe. Compared to other prognostic factors, IIPI exhibited better ability to predict overall survival. The multivariate analysis indicated that cirrhosis, differentiation, hilar invasion and IIPI were independent prognostic factors for ICC patients. An IIPI-based nomogram was also established and could predict the overall survival. In addition, the subgroup analyses based on clinical prognostic factors showed that the IIPI exhibited excellent prognostic influence. IIPI model is suitable for predicting the prognosis of postoperative ICC patients. Further research is needed to explore the relationship between postoperative recurrence and metastasis of ICC patients and IIPI.

## Introduction

Intrahepatic cholangiocarcinoma (ICC) is the second most common malignant liver tumor and poses a significant threat to patients^1^. Reports indicate that ICC accounts for 20% of liver malignancies and its incidence is increasing annually^2^. Though medical technology has advanced, surgical excision remains the primary method of treating ICC. Unfortunately, the prognosis for patients is bleak, with a 5-year survival rate of only 35% at best^3^. Numerous studies have identified potential prognostic biomarkers of ICC patients. However, the practical clinical application of these biomarkers remains limited^4–6^. In order to predict the prognosis of ICC patients, it is important to identify a biomarker that has both high value and convenient application in clinical practice.

It was reported that inflammation may contribute significantly to tumor development. Various ratios, including neutrophil–lymphocyte ratio (NLR), platelet-lymphocyte ratio (PLR), and lymphocyte-monocyte ratio (LMR), have been identified as potential indicators of this relationship^7,8^. Recent studies have shown that the lung immune prognostic index (LIPI), which consists of dNLR and lactate dehydrogenase (LDH) can be used as a predictive tool for immunotherapy response in lung cancer. This is due to its ability to reflect the proinflammatory state, which also determines the prognosis of small cell lung cancer^9^. LIPI, which stands for the liver immune-inflammatory panel, is not only related to targeted therapy and immunotherapy to a certain extent, but also has a correlation with irAE^10^. These studies all indicate the powerful predictive power of LIPI.

The prognosis of ICC patients is poor and there are challenges in using clinical prediction models to evaluate their prognosis and determine the appropriate treatment. Currently, there is no research on ICC patients that examines the relationship between LIPI and lung cancer. We developed a prognostic model for ICC patients and assessed its prognostic ability.

## Patients and methods

### Patients

The clinical data of ICC patients from January 2015 to January 2023 in the department of liver surgery & liver transplant center of West China Hospital was retrospectively reviewed with the approval of institutional review board. And all methods were performed in accordance with the relevant guidelines and regulations. The inclusion criteria were as follow: (1) patients diagnosed as ICC with primary postoperative biopsy confirmation; (2) patients who presented complete hematological test results within 7 days before surgery, (3) patients with reliable follow up data and clinicopathological information. Exclusion criteria: (1) patients who had undergone previous treatment such as radiofrequency ablation, transarterial chemoembolization, or chemotherapy prior to hepatectomy; (2) patients who underwent surgical resection due to tumor rupture; (3) patients with other malignancies; (4) patients with hematological diseases. Overall, 389 ICC patients who met the criteria were included and each of them were followed up regularly till January 2023. All patients had outpatient follow-up appointments every 3 months for the first 2 years after surgery, every 6 months between 2 and 5 years after surgery, and once a year after 5 years, unless in cases of emergency.

### Data collection

Leukocytes count (Leut#), neutrophil count (Neu#), lymphocyte count (LYMPH#), monocytes count (MONO#), LDH, α-fetoprotein (AFP), carbohydrate antigen199 (CA199) and carcinoembryonic antigen (CEA) were extracted from the blood routine of the 389 patients prior to surgical treatment. The formulas for calculating NLR, LMR, and dNLR are as follows: NLR = Neut#/LYMPH#, LMR = LYMPH#/MONO#, and dNLR = Neut#/ (Leut#-Neut#).

In addition, gender, age, HBV infection status, cirrhosis status, tumor node metastasis (TNM) staging, degree of differentiation, number of tumor sites, microvascular invasion (MVI) status, and tumor metastasis status were collected from the patients’ medical records. OS was calculated from the date of diagnosis to the date of death or last follow-up. In the overall cohort, the optimal cutoff value for each hematological marker was calculated based on the time-dependent receiver operating characteristic (ROC) curve and converted into a binary variable according to the cutoff value.

### Establishment and validation of the IIPI

Referring to the development of the LIPI, we established the prognostic model IIPI by combining the hematological indexes with a higher area under curve (AUC) in the ROC curves according to our results. Then, we compared the prognostic predictive effect of the IIPI with that of other hematological factors and clinical characteristics by time-dependent ROC. To verify whether the IIPI is an independent predictive factor for ICC prognosis, we conducted univariate and multivariate analyses. Significant factors in univariate analyses were then subjected to multivariate analyses to determine independent risk factors for survival. Furthermore, the association between the IIPI and OS was also explored by Kaplan–Meier survival analysis.

### Construction and evaluation of the nomogram

After the above-mentioned screening process, the prognostic factors were used to construct a nomogram for predicting the OS. For each patient, the total point was equal to the sum of the points of all factors. The link between the total points and the probability of OS were shown at the bottom of the nomogram. The discrimination ability and accuracy of nomograms were evaluated by Harrell’s Concordance Index and calibration curve, respectively. The diagonal acts as a reference line and represents the best prediction. Decision curve analysis (DCA) was used to evaluate the clinical application of the nomogram by estimating the net benefits at different threshold probabilities. The clinical impact curve was also drawn to predict reduction intervention probability per 100 patients.

### Statistical analysis

Kolmogorov–Smirnov was used to assess whether continuous variables were normally distributed, and Mann–Whitney U test or Spearman correlation analysis was used to assess differences between continuous variables according to the results. Categorical variables were evaluated using the chi-square test and the fisher’s exact test based on the number of individuals in each group. All statistical analyses were conducted using R software, version 4.1.0 (Institute for Statistics and Mathematics, Vienna, Austria). *p* values < 0.05 were considered to indicate statistical significance.

### Ethical approval

Written informed consent for participation was not required for this study. The study is in accordance with the national legislation and the institutional requirements.

## Results

### Patient characteristics and optimal cut-off values of hematological factors

**Figure 1:**
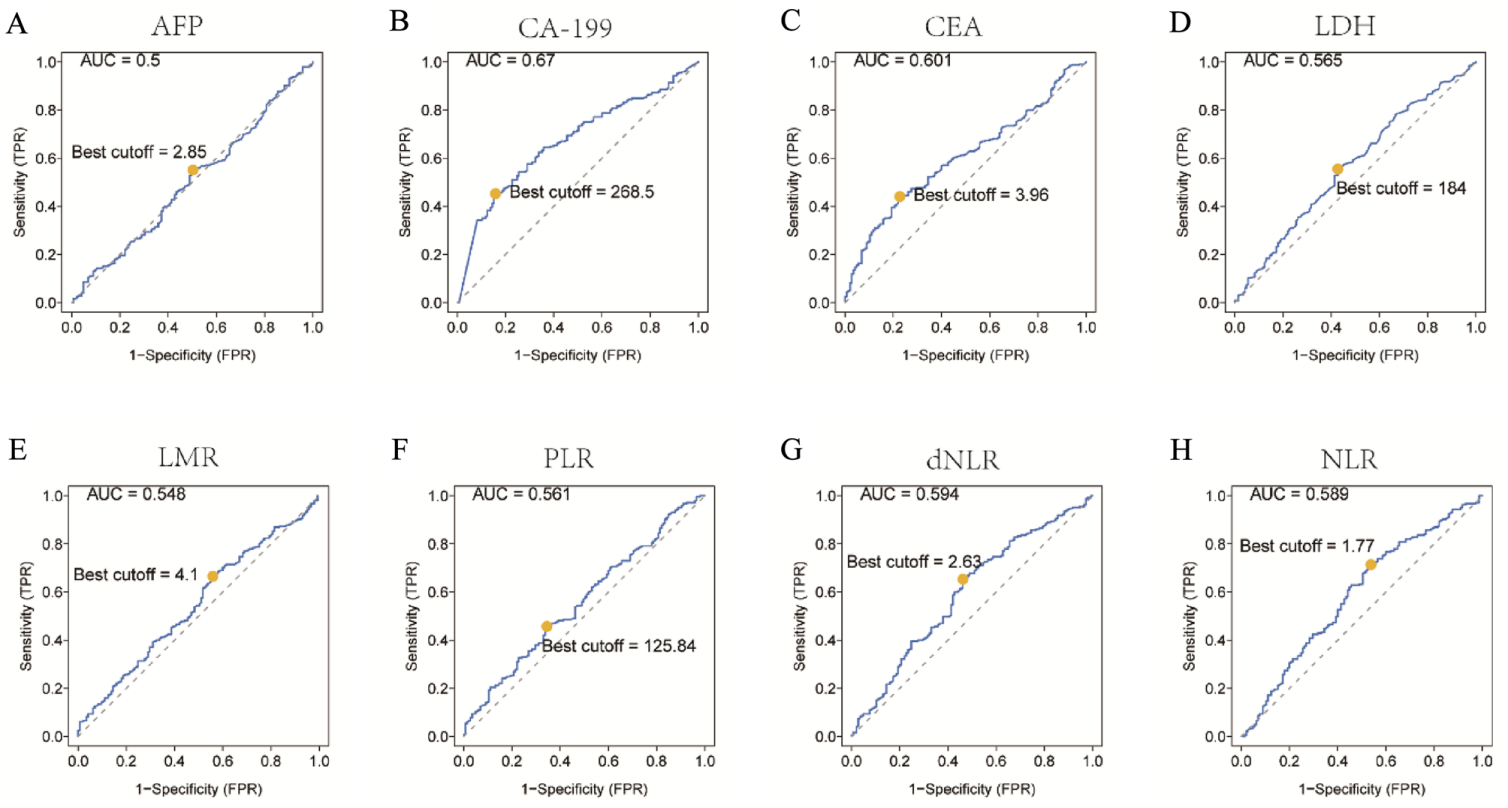
ROC analysis of different hematological biomarkers. (**A**–**H**) The AUC and best cutoff values of AFP, CA199, CEA, LDH, LMR, PLR, dNLR, NLR were shown, respectively. The vertical axis represents the sensitivity and the horizontal axis represents the 1-specificity. AFP, α-fetoprotein; CA199, Carbohydrate antigen199; CEA, carcinoembryonic antigen; dNLR, derived neutrophil-to-lymphocyte ratio; LDH, lactate dehydrogenase; LMR, lymphocyte-monocyte ratio; PLR, platelet–lymphocyte ratio; dNLR, derived neurtrophil to lymphocyte ratio.

A total of 389 ICC patients were obtained with an average age of 53 years. Among them, 200 were male and 189 were female (Table 1). According to the ROC analysis, the AUCs and optimal cuff-off of hematological markers including one of the factors of LIPI, dNLR were calculated. The AUCs and optimal cut-off were 0.5 and 2.85 for AFP, 0.67 and 268.5 for CA199, 0.601 and 3.96 for CEA, 0.565 and 184 for LDH, 0.548 and 4.1 for LMR, 0.561 and 125.84 for PLR, 0.594 and 2.63 for dNLR, 0.589 and 1.77 for NLR, respectively (Fig. 1). The results suggested that dNLR, CA199 and CEA were most appropriate factors for prognosis prediction analysis.

**Table 1:** Characteristics of 389 intrahepatic cholangiocarcinoma patients.

	Patients	IIPI				*p* value
		None	Light	Moderate	Severe	
Total patients	389	79	155	93	62	
Age (years)						0.020
> 60.0	176	31	77	49	19	
≤ 60.0	213	48	78	44	43	
Gender						0.779
Male	189	38	80	42	29	
Female	200	41	75	51	33	
HBV status						0.003
Positive	96	30	41	14	11	
Negative	293	49	114	79	51	
Number of lesions						0.084
Multiple	102	13	48	22	19	
Single	287	66	107	71	43	
MVI						0.531
Positive	46	6	22	11	7	
Negative	343	73	133	82	55	
TNM staging						0.101
I/II	95	25	42	17	11	
III/IV	294	54	113	76	51	
Cirrhosis						0.758
Positive	37	8	17	8	4	
Negative	352	71	138	85	58	
Differentiation						0.204
Medium/high	105	28	35	24	18	
Poor	284	51	120	69	44	
AFP (ng/ml)						0.120
> 7.0	50	9	24	6	11	
≤ 7.0	339	70	131	87	51	
CA199 (u/ml)						0.000
> 30.0	280	42	94	82	62	
≤ 30.0	109	37	61	11	0	
CEA (ng/ml)						0.000
> 5.0	169	3	46	58	62	
≤ 5.0	220	76	109	35	0	
dNLR						0.000
> 2.63	228	3	101	68	56	
≤ 2.63	161	76	54	25	6	
NLR						0.000
> 1.77	252	0	118	72	62	
≤ 1.77	137	79	37	21	0	
LDH						0.053
> 250	55	6	23	13	13	
≤ 250	324	73	132	80	39	
LMR						0.000
> 4.1	145	50	53	26	16	
≤ 4.1	244	29	102	67	46	

HBV, hepatitis B virus; MVI, microvascular invasion; TNM, Tumor Node Metastasis; AFP, α-fetoprotein; CA199, Carbohydrate antigen199; CEA, carcinoembryonic antigen; dNLR, derived neutrophil-to-lymphocyte ratio; LDH, lactate dehydrogenase; LMR, lymphocyte-monocyte ratio.

### Establishment of IIPI and survival analysis of various hematological factors

**Figure 2:**
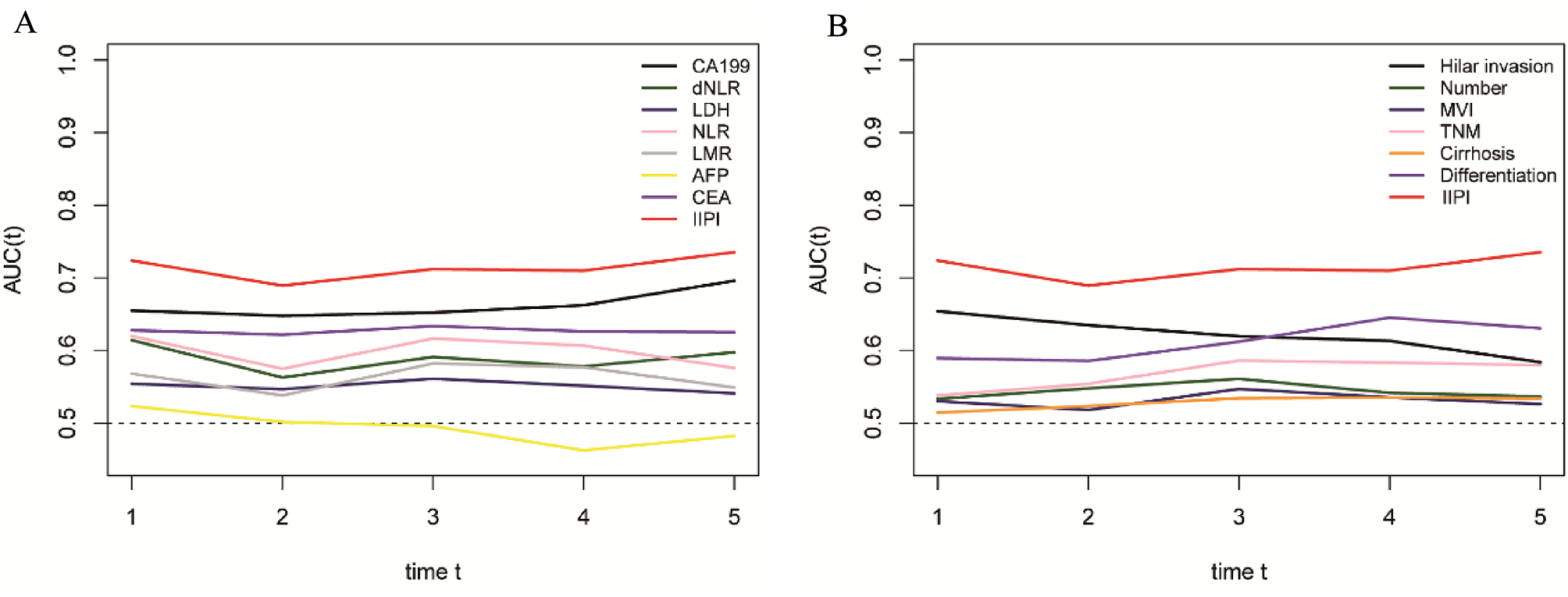
Comparison of different hematological and clinical biomarkers in predicting the overall survival of intrahepatic cholangiocarcinoma patients. (**A**) The difference in the predictive ability of different hematological markers is shown in the time-dependent ROC curve, in which a larger AUC value indicates a better predictive ability. (**B**) Difference in the predictive ability of different clinical features. CA199, Carbohydrate antigen199; dNLR, derived neutrophil-to-lymphocyte ratio; LDH, lactate dehydrogenase; LMR, lymphocyte-monocyte ratio; AFP, α-fetoprotein; CEA, carcinoembryonic antigen; IIPI, intrahepatic immune prognostic index; HI, hilar invasion; MVI, microvascular invasion; TNM, Tumor Node Metastasis.

After comparing the prognostic power of different hematological biomarkers, we found that dNLR, CA199 and CEA were more effective in predicting ICC prognosis than other single hematological factors. Therefore, by combining these three hematological factors, we developed a new prognostic model called IIPI (Fig. 2). As shown in Fig. 2, IIPI exhibited evident better prognostic ability than hematological and clinical factors, indicating that IIPI is a better predictor than existing prognostic factors. All the 389 ICC patients were divided into 4 groups according to the IIPI. The critical value of the three factors was determined by the ROC analysis, with 1.77, 268.5 and 3.96 for dNLR, CA199 and CEA respectively. All patients were grouped according the number of positive factors. 79 patients were assigned to group none with none of IIPI. 155 patients were assigned to group light with the presentation of one IIPI. 93 patients were assigned to group moderate with the presentation of two IIPIs and 62 patients were assigned to group severe with the presentation of three IIPIs.

### Univariate analysis and multivariate analysis

**Figure 3:**
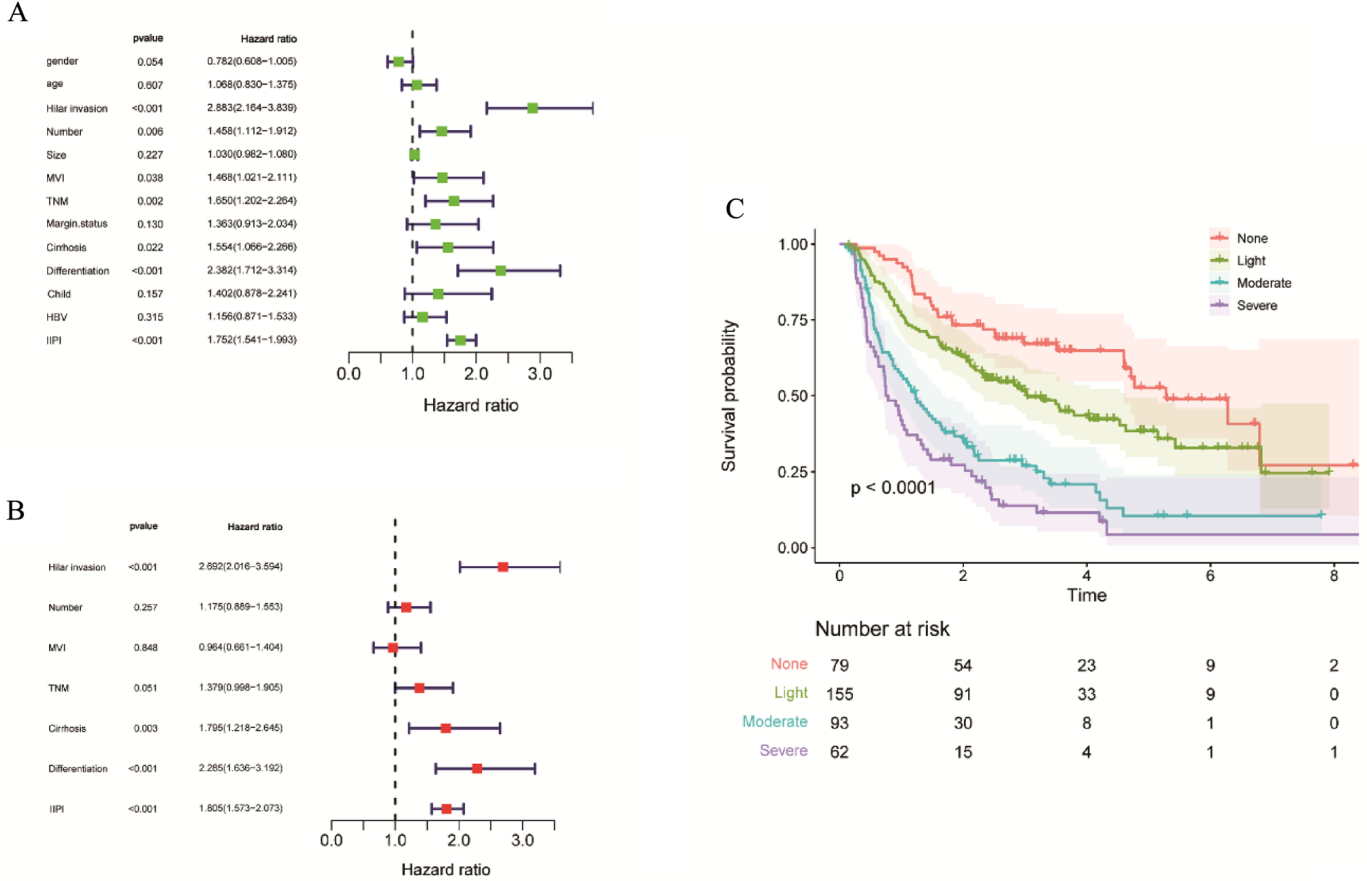
Independent risk factors of OS in 389 intrahepatic cholangiocarcinoma patients. (**A**) Univariate analysis of clinical characters. (**B**) Multivariate analysis of significant clinical characters in univariate analysis to determinate independent prognostic factors. (**C**) The overall survival comparison of different grades of IIPI. HI, hilar invasion; MVI, microvascular invasion; TNM, Tumor Node Metastasis; HBV, hepatitis B virus; IIPI, intrahepatic immune prognostic index.

The Univariate analysis suggested that the number of ICC lesions (HR = 1.458(1.112–1.912), *p* = 0.006), MVI (HR = 1.468(1.021–2.111), *p* = 0.038), TNM staging (HR = 1.650(1.202–2.264), *p* = 0.002), cirrhosis (HR = 1.554(1.066–2.266), *p* = 0.022), differentiation (HR = 2.382(1.712–3.314), *p* < 0.001), hilar invasion (HR = 2.883(2.164–3.839), *p* < 0.001) and IIPI (HR = 1.752(1.541–1.993), *p* < 0.001) were associated with OS (Fig. 3A). These factors were then subjected to multivariate analyses to further select independent prognostic factors. As shown in Fig. 3B, cirrhosis (HR = 1.795(1.218–2.645), *p* = 0.003), differentiation (HR = 2.285(1.636–3.192), *p* < 0.001), hilar invasion (HR = 2.692(2.016–3.594), p < 0.001) and IIPI (HR = 1.805(1.573–2.073), *p* < 0.001) were found to be independently predictive of prognosis. Furthermore, the OS among the 4 groups according to IIPI was compared and it was found that with the score being higher, the survival probability being lower significantly (Fig. 3C).

### Construction and validation of IIPI based nomogram

**Figure 4:**
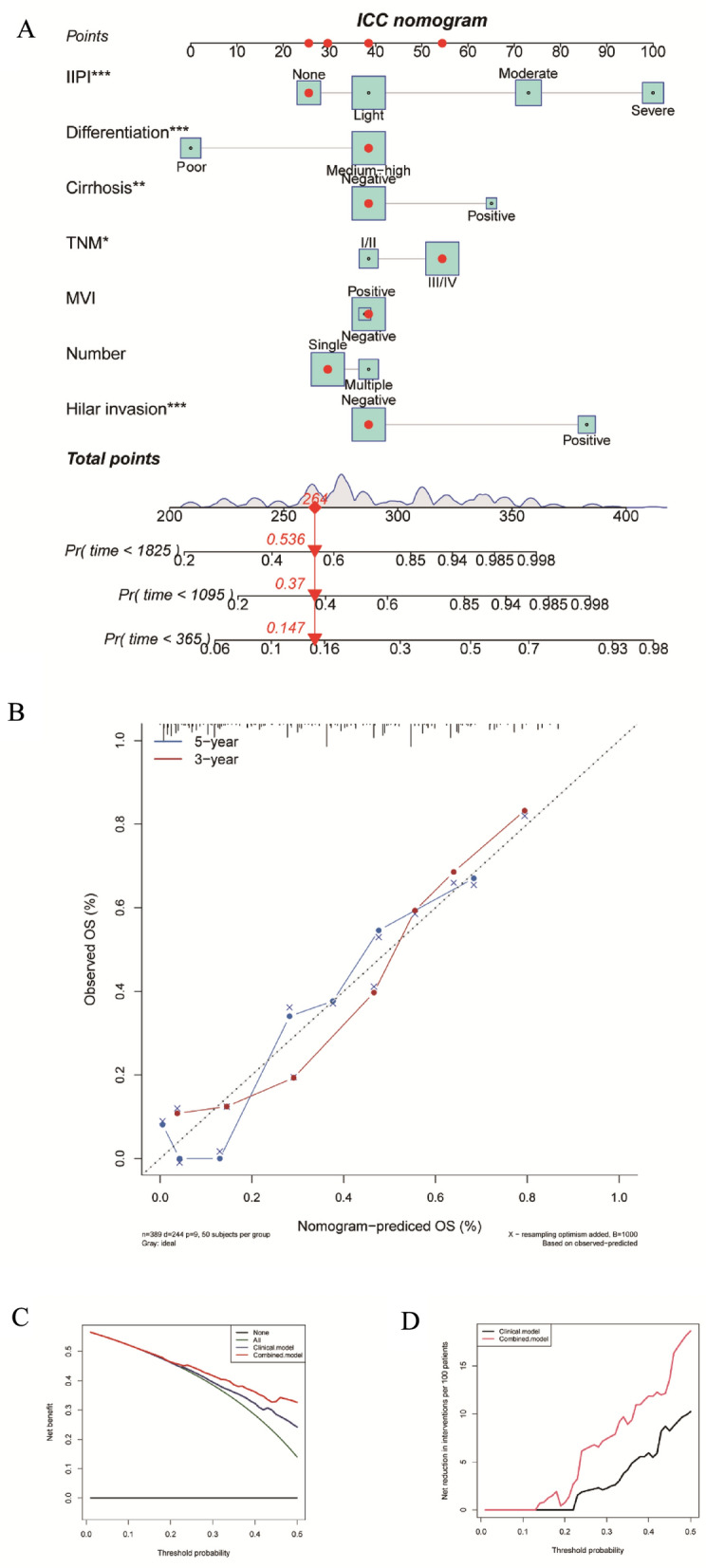
Construction and validation of the intrahepatic cholangiocarcinoma overall survival nomogram. (**A**) The nomogram was constructed by combing dNLR, CA199 and CEA and the sum of the scores for each covariate was the nomogram total score. (**B**–**D**) This nomogram was validated by the calibration curve, decision curve analysis, and clinical impact curve. IIPI, intrahepatic immune prognostic index; TNM, Tumor Node Metastasis; MVI, microvascular invasion; HI, hilar invasion; OS, overall survival.

To verify the clinical value of the IIPI, we constructed a nomogram combining the IIPI with clinical features. As shown in Fig. 4A, Cox proportional hazards regression assigned a score based on the hazard ratio for each covariate, and the sum of the scores for each covariate was the nomogram total score. The C-index of this ICC nomogram was 0.78, and the calibration curve indicated that this nomogram could accurately predict 3- and 5-year OS (Fig. 4B). Eventually, we also explored the clinical benefits and of this nomogram with clinical DCA (Fig. 4C,D). Our results demonstrate that the combined model (clinical features and IIPI) could bring significant net benefits over the model with only clinical features.

### Subgroup analyses based on clinical prognostic factors

**Figure 5:**
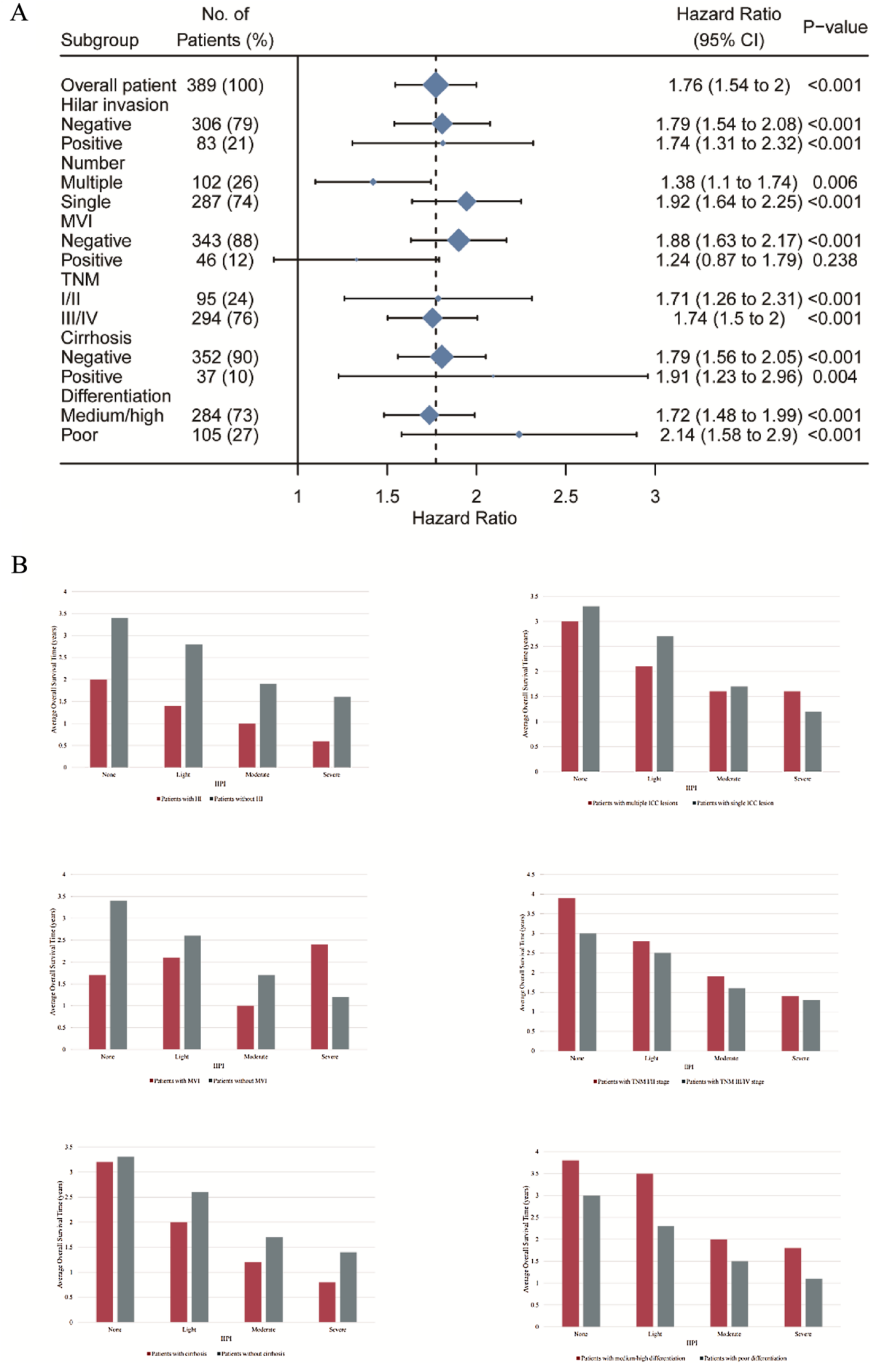
Subgroup analyses based on clinical prognostic factors. (**A**) Hazard Ratio of different IIPI groups for overall survival in different subgroups. (**B**) Histogram of average overall survival time comparison HI of different IIPI levels in different clinical feature subgroups. HI, hilar invasion; MVI, microvascular invasion; TNM, Tumor Node Metastasis; ICC, intrahepatic cholangiocarcinoma; IIPI, intrahepatic immune prognostic index.

In a further validation of IIPI, we conducted subgroup analyses based on prognostic factors to compare the OS of different IIPI degree patients. As seen in Fig. 5, the OS of four IIPI groups were not significantly different in patients with MVI. However, the OS of patients without MVI and with other clinical prognostic factors being positive or negative all reached statistical differences, indicating that IIPI could effectively predict OS no matter ICC patients with or without hilar invasion, cirrhosis, with single or multiple tumor lesions, with early or late TNM stage, with poor or medium and high differentiation. While its prognostic power for patients with MVI was not so good. Even though, the IIPI exhibited excellent prognostic influence.

## Discussion

ICC is a common form of liver cancer with a rising occurence^11,12^, representing 5%-20% of all liver malignancies. The inflammatory environment is believed to cause damage to DNA and stimulate the growth of bile duct cells, leading to a potentially harmful situation^13,14^. The prognosis for ICC patients is generally poor, with high recurrence rates even after surgical excision. As a result, it is crucial to investigate biomarkers that can aid in risk stratification and treatment guidance.

Numerous studies have reported that inflammation is a crucial factor in the development of various types of tumors^15,16^. Mezquita et al.^17–19^ introduced LIPI, a novel hematologic marker based on inflammatory indicators composed of dNLR and LDH, which plays a significant role in lung cancer. He et al. demonstrated creativity by developing OIPI, a predictor specifically designed for bone tumors. They combined the role of LIPI in lung cancer to create OIPI, which plays a crucial role in predicting the prognosis and metastasis of bone tumors^20,21^. To date, there has been no reports on the prognostic effect of LIPI in patients with ICC.

This study aimed to establish a prognostic model called IIPI for ICC patients after surgery, inspired by the crucial role of LIPI in predicting prognosis and guiding immunotherapy selection in lung cancer. The study applied factors in LIPI to ICC patients and found that LDH was not effective in predicting the prognosis of ICC (Fig. 1). In this study, CA-199 and CEA biomarkers were found to be still significant in predicting the prognosis of ICC, which is consistent with the findings of Moro et al^22^. The LIPI and CEA, CA199 were combined to develop IIPI. The results showed that IIPI had a higher prediction efficiency than other markers of hematology and clinical characteristics, as measured by ROC. After conducting both univariate and multivariate analysis, our findings indicate that IIPI can serve as an independent risk factor for predicting the prognosis of ICC patients. It was observed that the IIPI score was able to precisely and consistently reflect the prognosis of postoperative ICC patients. In addition, the survival analysis (Fig. 3 C) further supports this claim, as higher IIPI scores were associated with poorer prognosis.

Since its initial proposal, the nomogram graph has become a widely utilized tool in various studies, aiding clinicians in both diagnosis and treatment of patients^23^. Incorporating clinical characteristics and IIPI, we have developed an IIPI-based nomogram to predict the prognosis of ICC patients. By using individual information and corresponding values, a total score can be calculated to assess the risk of prognosis for patients. The study demonstrates the high accuracy of the IIPI-based nomogram graph in predicting 3 and 5-year metastasis rates in ICC patients. The IIPI-based nomogram graph was found to be more beneficial in prognosis predicting of postoperative ICC patients compared to the prediction model without IIPI, as confirmed by the DCA curve. Thus, the nomogram can be considered as a reliable predictor of prognosis in ICC patients.

Recent studies have focused on developing prognostic models for ICC patients. However, the traditional hematological indicators CEA and CA-199 have limited prognostic effects in predicting the prognosis of ICC patients^24,25^. In recent years, there has been increasing research on the role of inflammation in the development of tumors. As a result, many studies have been conducted to explore the use of inflammatory factors as a means of predicting tumor diagnosis and prognosis^26,27^. In a study analyzing data from 660 patients who underwent ICC hepatectomy, researchers developed the LabScore score by combining platelet count, CA19-9, albumin, and neutrophil to lymphocyte ratio (NLR) to predict ICC prognosis. A higher LabScore indicates a worse prognosis^28^. In addition, Qiu’s^29^ study highlights the significance of aspartate amination transaminase (AST) to lymphocyte ratio index and CA19-9 level in determining the prognosis of patients with intrahepatic cholangiocarcinoma (ICC). Qiang et al.^30^ conducted a study on 237 ICC patients who underwent routine resection and used immunohistochemistry to detect glypican-1 and glypican-3. The study found that high expression of glypican-1 and glypican-3 was associated with a poor prognosis. However, these prediction models often have their own specific limitations. Due to the lack of immune-related factors being combined, these studies showed a deficiency in the predicting the immune status of ICC patients which is crucial for evaluating the immunotherapies indication for poor prognostic ICC patients. In this study, we utilized the predictive effect of LIPI which is predictive of immune status, and applied it to ICC. The results demonstrated that the new predictive model, IIPI, had excellent efficacy in ICC patients and had the strongest sensitivity compared to other features. IIPI categorized postoperative ICC patients into four groups, where high IIPI scores were strongly associated with poorer outcomes.

However, there are several limitations in this study. Firstly, owing to the retrospective nature of the study, selection bias was possible. Secondly, the study only investigated the prognosis of ICC patients after surgical resection and did not explore the recurrence and metastasis of ICC patients, which are associated with the survival. Further research is needed to address these limitations. Thirdly, this study has limitations due to being a single-center study, which may result in a certain degree of bias as it does not cover a large number of patients or patient information from different institutions. Furthermore, this study did not investigate the association between IIPI and the immune status of ICC patients. In the future, large randomized prospective studies would overcome these limitations and explore the relation between IIPI and immune status of ICC patients.

## Conclusion

This study presents the IIPI model exhibited brilliant power in predicting the prognosis of postoperative ICC patients, which may help to identify poor prognostic ICC patients who could benefit from adjuvant therapies like radiotherapy, targeted therapy, and immunotherapy. This study suggests that patients with higher IIPI levels possess poorer outcomes and may benefit from the treatments discussed. Further research is needed to explore the relationship between postoperative recurrence and metastasis of ICC patients and IIPI.

## Data Availability

The datasets used and/or analyzed during the current study are available from the corresponding author on reasonable request.
